# Supplementary material for: Microplastic from beach sediment to tissue: a case study on burrowing crab Dotilla blanfordi
Source: PeerJ. 2024 Jul 12;12:e17738. doi: 10.7717/peerj.17738 (PMC11249004; doi:10.7717/peerj.17738)
Supplement: Supplemental Information 2 [file peerj-12-17738-s002.docx]

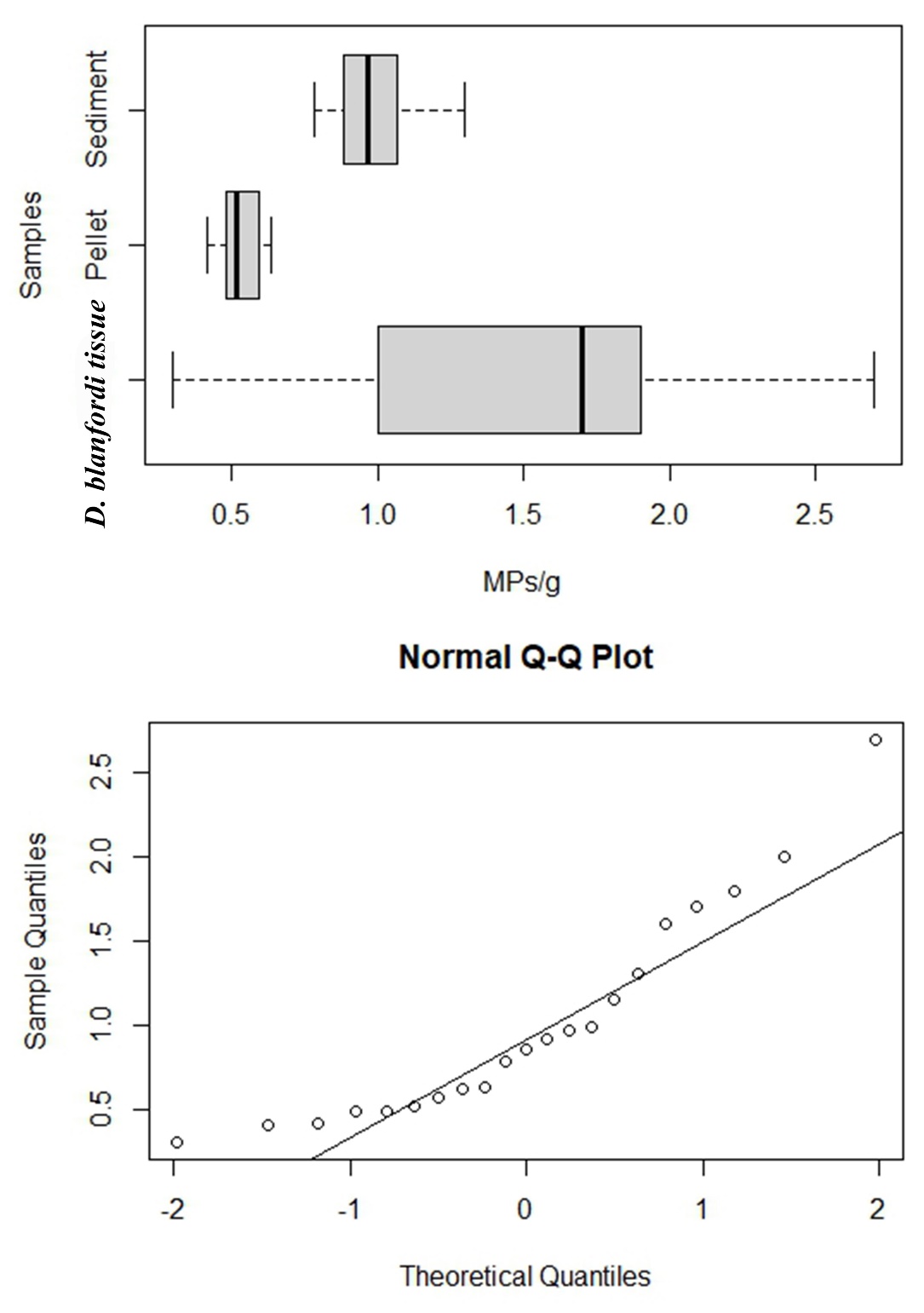


**Supplementary figure 1s:**

Abundance of microplastic contamination in the burrow sediment, feeding pellet and crab’ tissue collected from study site Asharmata, showing not a normal distribution (Shapiro-Wilk test, W = 0.88, p = 0.01).
